# Left main coronary artery to pulmonary artery fistula presenting as angina and ventricular tachycardia – A case report and literature review

**DOI:** 10.1002/ccr3.7231

**Published:** 2023-05-01

**Authors:** Simone Gong, Silvana Marasco, Michael Wong, Martin Hiscock

**Affiliations:** ^1^ Department of Cardiology Epworth Hospital Richmond Melbourne Victoria Australia; ^2^ Department of Medicine University of Melbourne Melbourne Victoria Australia; ^3^ CJOB Cardiothoracic Surgery Department The Alfred Hospital Melbourne Victoria Australia; ^4^ Department of Surgery Monash University Melbourne Victoria Australia

**Keywords:** angina, coronary artery fistula, coronary steal, left main ischemia, ventricular tachycardia

## Abstract

Coronary artery fistulae are an uncommon abnormality of the coronary arteries, but when hemodynamically significant can present as angina, dyspnea, and arrhythmia as a rare cause of functional myocardial ischemia via ‘coronary steal phenomenon’.

## Graphical abstract

Coronary angiogram depicting coronary artery fistula coursing from left main to pulmonary trunk, which resulted in functional myocardial ischemia.

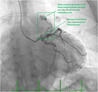

## 1 INTRODUCTION

Coronary artery fistulae (CAFs) are rare, largely congenital, anomalies of the coronary anatomy which involve the presence of abnormal connections between the coronary arteries and the great vessels or the chambers of the heart. In children, they are commonly asymptomatic and found incidentally. However, adults with hemodynamically significant coronary fistulae will often present with symptoms and life‐threatening complications such as myocardial ischemia or infarction, aneurysm formation and rupture, arrhythmias, and congestive cardiac failure. As a result, closure is indicated for large or symptomatic fistulae, either with surgical or percutaneous techniques.

We present here a case of a 51‐year‐old male who was found to have a left main coronary to pulmonary artery fistula with aneurysmal dilatation following presentation with angina, dyspnea, and ventricular tachycardia. He subsequently underwent successful surgical ligation of the fistula.

## 2 CASE REPORT

A 51‐year‐old gentleman was referred for investigation of unstable angina, dyspnea on exertion, and bouts of tachycardia, which was diagnosed on Holter report to be broad complex ventricular tachycardia. During the 24‐h Holter monitoring, he had four separate runs of ventricular tachycardia, the longest of which was 477 beats at a rate of 175 bpm, and coincided with the patient feeling dizzy and lethargic. He is otherwise healthy and has no cardiovascular risk factors. His only history of note was recurrent deep vein thrombosis provoked by soft tissue injury, with one episode of pulmonary embolus.

On examination, he was normotensive and in sinus rhythm. On precordial auscultation, he had no cardiac murmurs or added sounds. Laboratory tests, including cardiac enzymes, were normal other than a positive D‐dimer test.

**FIGURE 1:**
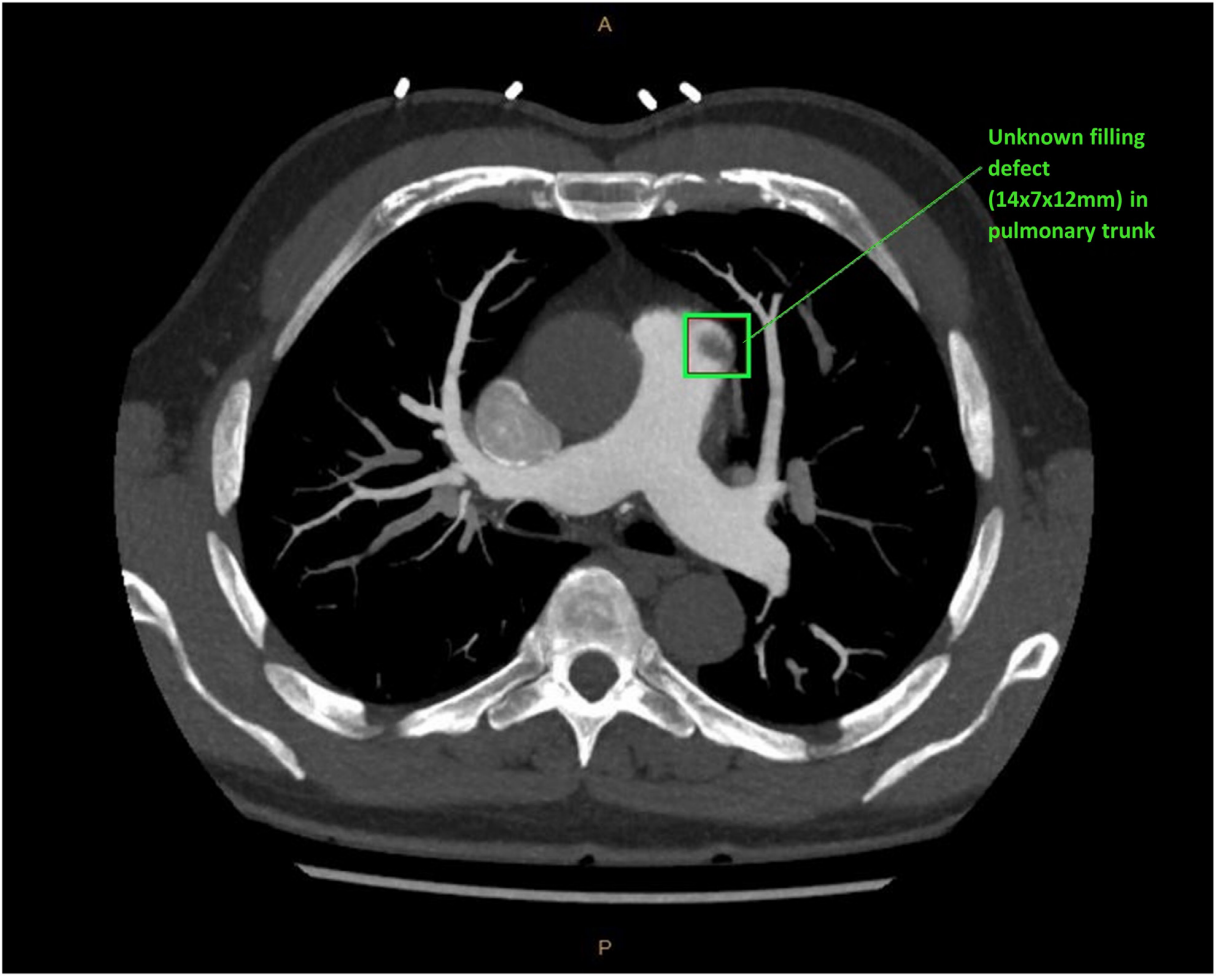
CTPA depicts an unknown filling defect in the pulmonary trunk region. CTPA, CT pulmonary angiogram.

A CT pulmonary angiogram (CTPA) was therefore performed, particularly given the history of pulmonary embolus. Whilst negative for pulmonary embolus, a filling defect of unknown cause and significance was seen in the pulmonary trunk region at the superolateral aspect, measuring 14 × 7 × 12 mm (Figure 1).

The plain chest film was unremarkable, demonstrating a normal cardio‐mediastinal silhouette. Transthoracic echocardiography (TTE) revealed normal left ventricular function (LVEF 65%), moderate bi‐atrial enlargement, and a mildly dilated right ventricle. The main pulmonary artery could not be visualized adequately in this study.

The patient's ECG was initially normal, but during an episode of chest pain during his admission, his ECG showed ST elevation in lead aVR and widespread anterolateral depression, consistent with left main stem coronary artery ischemia.

**FIGURE 2:**
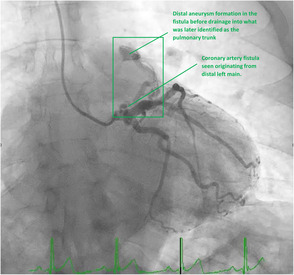
Coronary angiogram depicting fistula likely originating from the left main coronary artery with distal aneurysmal dilatation.

A subsequent coronary angiogram revealed the coronary arteries were free of significant disease but angiography of the left coronary system revealed a CAF with distal left main stem aneurysm formation. The exact location of the fistula drainage could not be confirmed via angiography. TIMI II flow down the Left Anterior Descending and Left Circumflex coronary arteries were apparent, suggesting a coronary steal phenomenon and likely explaining the patient's symptoms of functional myocardial ischemia (Figure 2).

**FIGURE 3:**
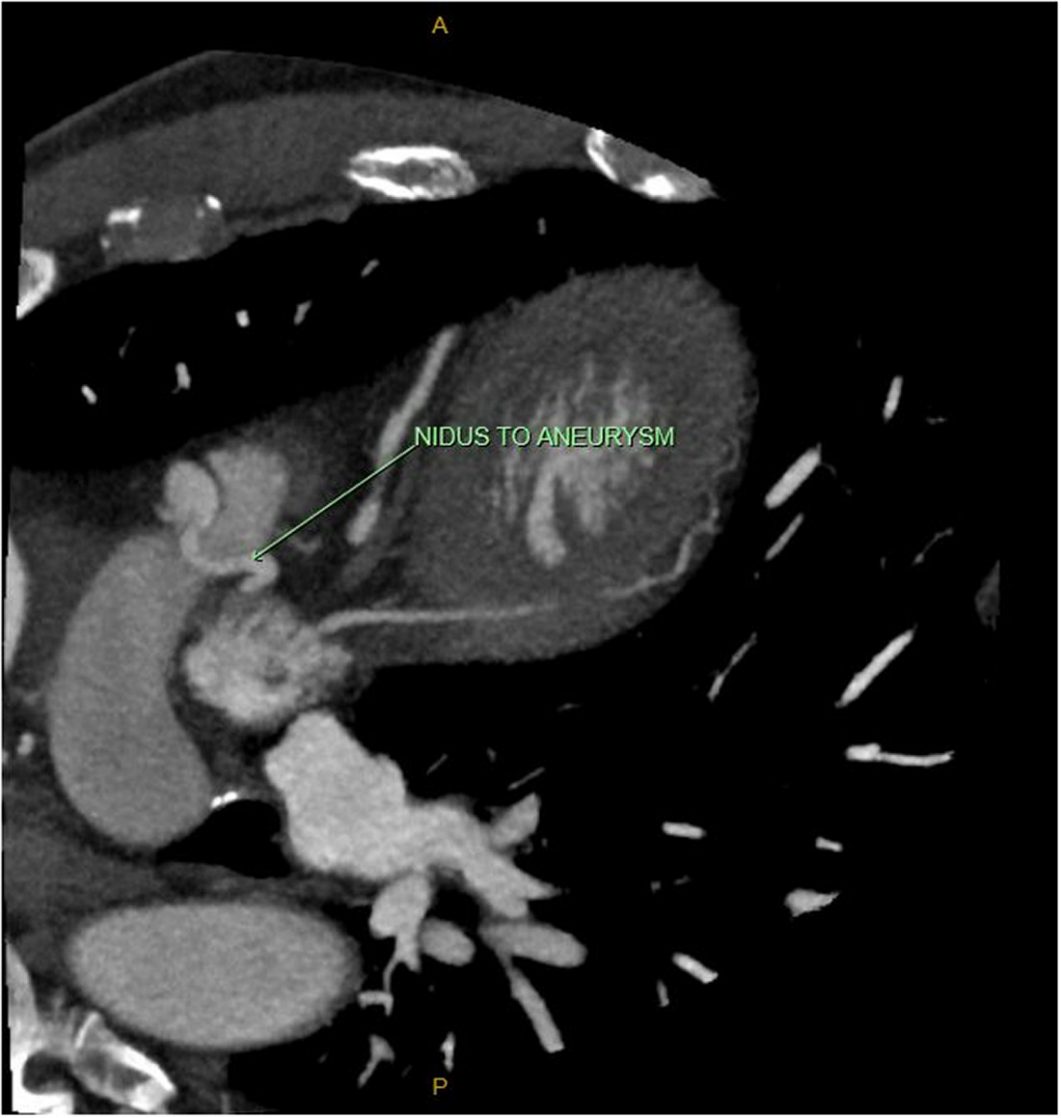
CTCA depicting vascular nidus of the fistula dilating into a focal 12 × 11 mm aneurysm in its distal segment. CTCA, CT coronary angiogram.

To confirm the exact course of the fistula, the patient underwent a CT coronary angiogram (CTCA). The fistula was found to course from the distal left main coronary artery to the pulmonary outflow tract. There were multiple, small feeding vessels arising from the left coronary artery coursing superiorly to form a small nidus and becoming aneurysmal distally, before emptying into the pulmonary artery (Figure 3). The aneurysm was focal, measuring 12 × 11 mm. The drainage of this fistula into the pulmonary trunk also explained the abnormality seen on CTPA.

Although the fistula was not observed on TTE during this admission, upon further review of the patient's record, we were able to see that it had been imaged in a TTE performed in 2011. Color flow Doppler examination revealed a flow disturbance characterized by a low‐velocity color jet just proximal to the pulmonary artery bifurcation. It was more visible in diastole, but continuous wave Doppler interrogation revealed this to be a continuous systolic‐diastolic flow disturbance. In 2011, the fistula was a smaller and without aneurysmal dilatation.

Given the patient presented with broad complex ventricular tachycardia, a cardiac MRI was also arranged to eliminate structural and infiltrative disease that may be responsible for the arrhythmia. The MRI was essentially unremarkable and it supported the findings from echocardiography, recording moderate bi‐atrial enlargement and LV and RV cavity sizes at the upper limits of normal, with normal systolic function. The Qp/Qs was 1.0. The Cardiac Index was 2.9 L/min/m^2^.

Since the patient was symptomatic with a life‐threatening arrhythmia and the coronary fistula was aneurysmal, he was referred for surgical closure. The procedure was minor and cardiopulmonary bypass was not needed. A small left anterior thoracotomy was performed through the second intercostal space and the aneurysm was identified directly below the incision over the pulmonary arterial trunk. It was oversewn and obliterated with sutures. An ultrasound probe was used to confirm no residual flow through the fistula before the closure of the pericardium.

The patient was discharged home 5 days after the operation. He reported no further chest pain and there was no recurrence of ventricular tachycardia on monitoring.

## 3 DISCUSSION

A CAF is a rare anomaly of the coronary anatomy which is estimated to affect 0.002% of the general population.^1^ They are most commonly congenital in origin but they can also be acquired from chest trauma, infection, or medical procedures such as surgery or cardiac catheterization.

Coronary artery fistulae can originate from either the right or the left coronary artery, with origin from the right coronary artery being slightly more common. In rare cases, the fistula involves more than one coronary artery.^2^, ^3^, ^4^ When from the left coronary artery, the fistula usually arises from the left anterior descending or left circumflex artery. CAFs occurring directly from the left main coronary artery are reported in fewer than 5% of cases.^5^ With regards to drainage, over 90% of fistulae drain into the right side of the heart, most often into the right ventricle, with about 15% draining into the pulmonary artery.^2^, ^3^, ^4^

The clinical presentation of CAFs can vary from asymptomatic to sudden cardiac death. Younger patients and those with small fistula size are usually asymptomatic. The most common presenting symptoms are exertional dyspnea and angina as a result of coronary steal phenomenon. However, patients can also present with unstable angina, syncope, arrhythmias, myocardial infarction, and heart failure, usually as a result of increased left to right shunt. More severe presentations often occur when there is concomitant coronary artery disease or Left Ventricular dysfunction.^1^, ^3^, ^6^, ^7^ CAFs commonly become aneurysmal, with literature placing the incidence between 19% and 30%.^8^, ^9^ This increases the risk of infective endocarditis and can lead to mural thrombus, rupture, or side branch occlusion. In rare cases, complications such as hemopericardium and death may occur. Aneurysmal dilatation tends to occur with time, as seen in our patient.

On routine physical examination, an atypical systolic, diastolic, or continuous ‘machinery’ murmur, not unlike that heard in patent ductus arteriosus, is the most common sign of a CAF, occurring in a reported 37% of cases for coronary/pulmonary fistulae.^9^ Although precise auscultation can provide information regarding the site and drainage of the fistula when a murmur can be heard, further investigations are required to establish diagnosis.

Coronary angiography can be used to diagnose a CAF, but CTCA is the current gold standard as it is better able to identify the precise course of a fistula(e). As demonstrated in this case, color Doppler echocardiography may be helpful in identifying a fistula and establishing the clinical severity of the fistula through assessment of resulting structural changes of the heart such as chamber enlargement and dysfunction and measurement of Qp/Qs. Transesophageal echocardiography (TOE) provides better visualization of a CAF than TTE and is also a valuable tool for intra‐operative cardiac monitoring.^10^

The management of CAFs is dependent on their size and symptomatology. The European Society of Cardiology (ESC) and American Heart Association (ACC/AHA) recommend that small, asymptomatic CAF can be treated conservatively and monitored with echocardiograms every 3–5 years in case of enlargement, degeneration, or symptomatic progression. Larger fistulae have a higher risk of complications and should be closed either percutaneously or surgically, even if asymptomatic. Small to moderate fistulae should only be closed if symptomatic or in the presence of complications such as unexplained structural changes or dysfunction seen on echocardiography, a Qp/Qs exceeding 1.5:1, or aneurysmal degeneration.^11^, ^12^, ^13^

If closure is necessary, CAF morphology must be considered. Fistulae which have a tortuous pathway morphology and multiple feeding vessels should be treated surgically. The overall operative mortality rate for surgical treatment is approximately 2% in studies at The Texas Heart Institute,^14^ which could be further reduced with less invasive approaches such as thoracotomies. Percutaneous closure devices are only recommended in select cases, when the fistula has simple morphology: an accessible, proximal origin, no branch vessels, and a distal narrowing to avoid embolism to the drainage site. Choice of interventional device is dependent on the anatomical characteristics of the fistula. For example, the Amplatzer Duct occluder or double umbrella devices can be used for larger fistulae, whereas covered stents, detachable balloons, microcoils, and histoacryl resin have all successfully closed smaller fistulae. Coils are used most commonly because of their smaller sheath and catheter delivery size, as well as the lower cost.^15^ Armsby et al. reviewed the outcomes of transcatheter closure in 45 patients, where there was a successful occlusion rate of 82%. One of the main complications of transcatheter intervention is device embolization. This study included four patients with device embolization to tricuspid valve or distal pulmonary artery, and one patient died where the coil was displaced from his large fistula into the left main coronary and subsequently dissected the artery.^16^ It is because of this possibility that percutaneous closure is also not recommended for aneurysmal or large high‐flow fistulae to prevent coil displacement.^17^, ^18^

Given that CAFs can recur or recanalize, with literature citing rates of up to 25%,^1^, ^17^ all patients with a history of CAFs should be followed up with regular echocardiography.

## 4 CONCLUSION

Coronary artery fistulae can be an unusual cause of angina and dyspnea associated with cardiac pathology. When found, they should be monitored and closure considered to prevent patient complications. Patients who undergo either percutaneous or surgical closure of a CAF have good prognosis, with normal life expectancy.

## AUTHOR CONTRIBUTIONS

**Simone Gong:** Conceptualization; data curation; formal analysis; visualization; writing – original draft; writing – review and editing. **Silvana Marasco:** Project administration; supervision; writing – review and editing. **Michael Wong:** Writing – review and editing. **Martin Hiscock:** Conceptualization; project administration; supervision; writing – review and editing.

## FUNDING INFORMATION

This research did not receive any specific grant from funding agencies in the public, commercial, or not‐for‐profit sectors.

## CONFLICT OF INTEREST STATEMENT

None.

## CONSENT

Written informed consent was obtained from the patient to publish this report in accordance with the journal's patient consent policy.

## Data Availability

Data sharing not applicable – no new data generated.
